# Tumor hijacks macrophages and microbiota through extracellular vesicles

**DOI:** 10.1002/EXP.20210144

**Published:** 2022-01-25

**Authors:** Jipeng Jiang, Jie Mei, Yongfu Ma, Shasha Jiang, Jian Zhang, Shaoqiong Yi, Changjiang Feng, Yang Liu, Ying Liu

**Affiliations:** ^1^ Postgraduate School Medical School of Chinese PLA Beijing P. R. China; ^2^ Department of Thoracic Surgery The First Medical Center of Chinese PLA General Hospital Beijing P. R. China; ^3^ CAS Key Laboratory for Biomedical Effects of Nanomaterials and Nanosafety & CAS Center for Excellence in Nanoscience National Center for Nanoscience and Technology of China Beijing P. R. China; ^4^ University of Chinese Academy of Science Beijing P. R. China; ^5^ GBA National Institute for Nanotechnology Innovation Guangdong P. R. China

**Keywords:** exosomes, extracellular vesicles, macrophage, microbiota, tumor microenvironment

## Abstract

The tumor microenvironment (TME) is a biological system with sophisticated constituents. In addition to tumor cells, tumor‐associated macrophages (TAMs) and microbiota are also dominant components. The phenotypic and functional changes of TAMs are widely considered to be related to most tumor progressions. The chronic colonization of pathogenic microbes and opportunistic pathogens accounts for the generation and development of tumors. As messengers of cell‐to‐cell communication, tumor‐derived extracellular vesicles (TDEVs) can transfer various malignant factors, regulating physiological and pathological changes in the recipients and affecting TAMs and microbes in the TME. Despite the new insights into tumorigenesis and progress brought by the above factors, the crosstalk among tumor cells, macrophages, and microbiota remain elusive, and few studies have focused on how TDEVs act as an intermediary. We reviewed how tumor cells recruit and domesticate macrophages and microbes through extracellular vehicles and how hijacked macrophages and microbiota interact with tumor‐promoting feedback, achieving a reciprocal coexistence under the TME and working together to facilitate tumor progression. It is significant to seek evidence to clarify those specific interactions and reveal therapeutic targets to curb tumor progression and improve prognosis.

## Graphical abstract

Tumor‐derived extracellular vesicles can carry malignant factors from tumor cells and help hijack macrophages and microbiota in the tumor microenvironment (TME). In turn, the hijacked macrophages and microbiota can respond with tumor‐promoting feedback, achieving a reciprocal coexistence in the TME and working together to facilitate tumor progression.

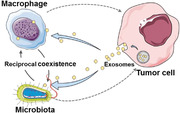

## 1 INTRODUCTION

Cancer poses a severe threat to public health and human natural life span. Cancer's increasing morbidity and mortality have spawned numerous studies on mechanism exploration and therapy investigation.^[^ ^1^ ^]^ Accumulating evidence has suggested that solid tumor is not just an aggregation of tumor cells but a microenvironment with dynamic exchanges of bioparticles among tumor cells, stromal cells, immune cells, and other constitutes.

Tumor‐associated macrophages (TAMs) are one of the main components in solid tumors; they are involved in nearly all types of cancers and play a critical role in the initiation and progression of carcinogenesis.^[^ ^2^ ^]^ In phenotype, TAMs are defined as M1 and M2 subtypes. M1 can secrete pro‐inflammatory cytokines (interferon (IFN) and interleukin (IL)), clear pathogens, and tumor cells through the type I immune response.^[^ ^3^ ^]^ M2‐like TAMs are involved in tissue repair, anti‐inflammatory response, and tumor progression.^[^ ^4^ ^]^

Some commensal microbes can also have a long‐term settlement in the tumor microenvironment (TME), inducing chronic inflammation and carcinogenesis.^[^ ^5^, ^6^ ^]^ *Helicobacter pylori*, *hepatitis B*, *schistosomiasis* are recognized as tumor‐inducing factors,^[^ ^7^ ^]^ *Escherichia coli*, *Bacteroides fragilis*, and *Fusobacterium nucleatum* also increase the risks of tumorigenesis.^[^ ^8^, ^9^ ^]^

Extracellular vehicles (EVs) are nanoscale membrane particles released by cells, exosomes, microvesicles, and apoptotic bodies are the most known EVs that are increasingly being recognized as biomarkers for many diseases, especially cancer.^[^ ^10^ ^]^ Cancer cells can secrete abundant EVs into the local microenvironment and circulatory system and play a crucial role in cell communication during tumor development. Tumor‐derived EVs (TDEVs) can carry a substantial number of cargos.^[^ ^11^ ^]^ mRNA, microRNA (miRNA), DNA, non‐coding RNA, proteins, and inorganic substances in the TDEVs can enter the host cells through various internalization pathways to affect gene transcription and interfere with the signaling pathways of host cells. Therefore, the malignant properties of a tumor may spread in the microenvironment and lead to further tumor progression.

**FIGURE 1:**
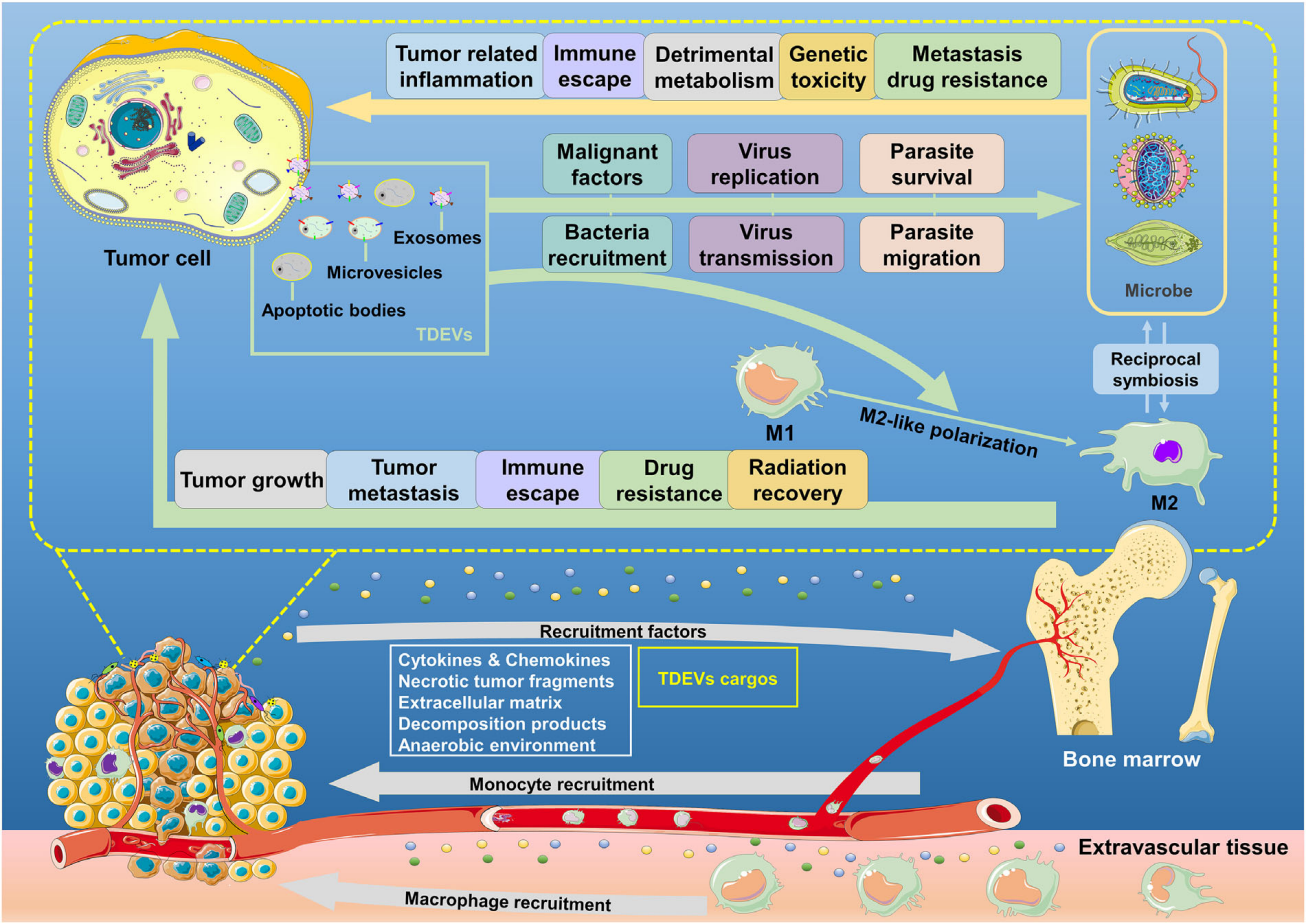
Interactions among tumor cells, macrophages, and microbiota in the tumor microenvironment. Tumor cells can secret substantial factors like EVs, chemokines, and cytokines, promoting the recruitment of monocytes and macrophages to the tumor microenvironment. After recruitment, the tumor‐derived factors can induce the M2‐like polarization of macrophages to tumor‐associated macrophages (M2‐TAMs); in return, M2‐TAMs will prompt tumor growth and metastasis, inhibit the tumor‐related immune response, and facilitate drug resistance and radiation recovery. Malignant collaborations also occur between tumor cells and microbiota. First, tumor‐derived factors can recruit and transfer malignant characteristics to microbiota, guarantee survival and migration, and promote their replication and transmission. Reciprocally, microbiota may cause tumor‐associated inflammatory, immunosuppression effects, detrimental metabolism, and genetic toxicity, inducing tumor metastasis and drug resistance. Besides, the capabilities of phagocytosis, antigen‐presenting, and digesting of macrophages may be deprived of in the tumor microenvironment, which will reduce the elimination of microbiota. Long‐term survival of microbiota can promote the phenotypic modulation of macrophages and generate the metabolic alteration of them

Chronic inflammation results from microbiome disruption and TAMs infiltration, which are two core factors in the TME. Until recently, the relationship between tumors and TAMs has not been fully elaborated. In addition, it is not clear how the microbiota communicates with the surroundings.^[^ ^7^ ^]^ An increasing body of evidence suggests that TDEVs may enter host cells in the TME. Malignant EVs can also be released from TAMs and microbiota in cell interactions.^[^ ^7^, ^12^ ^]^ For a long time, the intermediary role of EVs in tumors, TAMs, and the microbiome have been ignored. This review aims to further illustrate this unique and vital relationship by collecting evidence of tumor secreting EVs hijacking TAMs and the microbiome, followed by malignant feedbacks from TAMs and the microbiome to tumors (Figure 1). Target intervention against TAMs, pathogenic microbiomes, and TDEVs can be a promising strategy for tumor therapy.

## 2 TUMOR AND MACROPHAGES

### 2.1 The plasticity of macrophages and tumor fate

M1 and M2‐like TAMs have different characteristics and functions (Table 1). The phenotype of macrophages is closely related to tumor development.^[^ ^13^ ^]^ Classically, macrophages can be divided into M1 and M2‐like phenotypes; M1‐like TAMs can release a variety of pro‐inflammatory factors, immune activators, and chemokines. They can exert anti‐tumor effects through acute pro‐inflammatory response, immune activation, and phagocytosis. Generally, M1‐like TAMs can recognize tumor cells via surface markers, secret nitric oxide (NO), and reactive oxygen species (ROS) and directly kill tumor cells.^[^ ^14^, ^15^ ^]^ Besides, M1‐like TMAs can kill tumor cells by antibody‐dependent cell‐mediated cytotoxicity; during this process, the M1‐like TAMs can be activated and generate a non‐phagocytic killing effect after the binding of Fc receptors on the macrophages with the Fc segment of the antibody on the tumor cells.^[^ ^16^ ^]^ Moreover, M1‐like TAMs can eliminate tumor cells by presenting antigens to T cells, activating the specific immune response, regulating and promoting Th1 immune response. On the contrary, M2‐like TAMs can inhibit T cells' proliferation and activation, promoting the Th2 immune response. They can promote tumor cell proliferation via the secretion of IL‐6/8/10 and suppression of NO release and the iNOS pathway. M2‐like TAMs can also produce vascular endothelial growth factor (VEGF), platelet‐derived growth factor (PDGF), IL‐17, MMP2/9 and hence enhance the angiogenesis tumor invasion and metastasis.^[^ ^17^ ^]^

**TABLE 1:** The comparison of M1‐like TAMs and M2‐like TAMs

	**M1**	**M2**
Activating factors	LPS, IFN‐γ, GM‐CSF	TGF‐β, IL‐1β/4/6/10/13, TLR agonists
Marker expression	IL‐1R, TLR2/4CD68/80/86, iNOS, SOCS3, MHC II	Arg‐1, Ym1/2, CD86/163/200R/206, TLR‐1/8, MHC II, Fizz1, MMP2/3/7/9
Chemokines and cytokines expression	TNF‐α, IL‐1β, IL‐6/12/18/23, CCL2/3/5/8/9/10/11	IL‐1/6/10/12, TNF‐α, TGF‐β, CCL1/5/17/22/24, CCR2, CXCL10/16
Involved biological processes	Pro‐inflammatory response Th1 immune response tumor inhibition	Angiogenesis tissue repair Th1 immune response anti‐inflammatory reaction

### 2.2 Tumors recruit macrophages through extracellular vehicles

**FIGURE 2:**
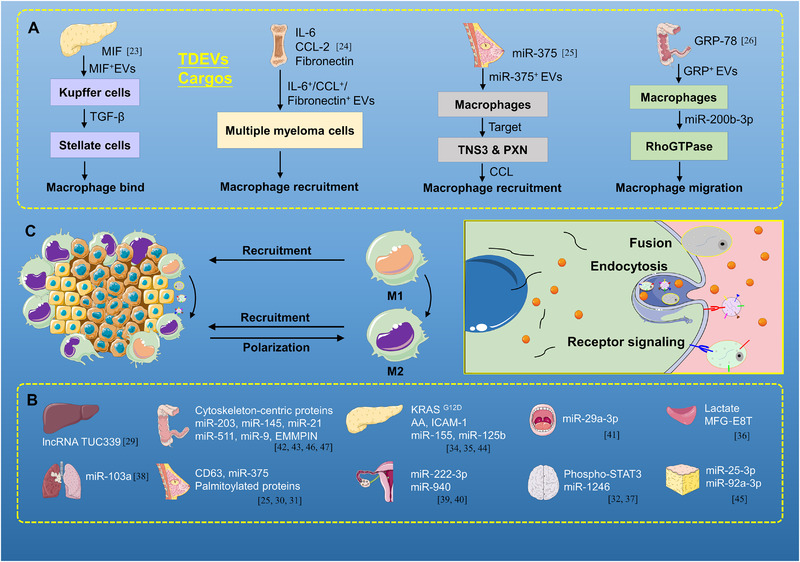
Tumor cells recruit and promote the phenotype transformation of macrophages. (A) Factors like cargos embedded in EVs, chemokines, and cytokines are involved in macrophage recruitment. For instance, MIF in pancreatic cancer, IL‐6, CCL‐2, fibronectin in myeloma, miR‐375 in breast cancer, and GRP‐78 in colon cancer. (B,C) In different cancers, tumor cells can transform macrophages into the M2‐like phenotype through TDEVs' cargos. Factors like cargos embedded in EVs, chemokines, and cytokines can act on different targets in the process of phenotypic transformation of macrophage, such as, lncRNA TUC339 can promote the macrophages to M2‐like phenotype

The microenvironment of tumors can release many chemo‐attractants to induce the recruitment of distant monocytes.^[^ ^18^ ^]^ Traditionally, the macrophages can be recruited into the TME under the stimulation of chemokines (chemokine ligand (CCL) 2/3/5/7/8/13/17/22) and cytokines (colony‐stimulating factor (CSF)‐1, VEGF, macrophage colony‐stimulating factor (M‐CSF), PDGF, IL‐6, IL‐8, tumor necrosis factor (TNF)‐α, transforming growth factor (TGF)‐β),^[^ ^19^, ^20^ ^]^ necrotic cell fragments, extracellular matrix, anaerobic environment.^[^ ^21^ ^]^ Decomposition products of the tumors also contribute to the recruitment of macrophages.^[^ ^22^ ^]^ Besides, the cargos embedded in the TDEVs have also been found to be directly or indirectly involved in macrophage recruitment. The exosomes derived from pancreatic ductal adenocarcinomas (PDAC) are rich in macrophage migration inhibitory factors. The uptake of these exosomes by Kupffer cells results in a rise in the production of fibronectin and TGF‐β secretion in hepatic stellate cells. After that, the recruiting capacity of bone marrow‐derived macrophages is improved significantly in the fibrotic microenvironment.^[^ ^23^ ^]^ Myeloma stromal cell‐derived exosomes contain abundant factors like fibronectin, CCL2, and interleukin‐6 (IL‐6), which will contribute to the recruitment of macrophages.^[^ ^24^ ^]^ Additionally, apoptotic breast cancer cell‐derived miRNA‐375 can strengthen the transportation of low‐density lipoprotein particles and upregulation of CCL2, which will enhance the recruitment of macrophages.^[^ ^25^ ^]^ Additionally, Glucose‐regulated protein 78 can also be transported to the extracellular space by exosomes and then activate the signaling pathway of fibronectin‐integrin‐β1‐ focal adhesion kinase MMP2/9. This promotes the adhesion between macrophages and matrix, along with the degradation of the extracellular matrix, to recruit macrophages^[^ ^26^, ^27^ ^]^ (Figure 2A).

### 2.3 Tumors remodel macrophages through extracellular vehicles

In the progression of many tumors, M1 is the main type in the early stage, while M2 dominates in the late stage. In this process, the disproportion M1/M2 TAMs may attribute to the components in TDEVs. Exosomal lactate in prostate cancer,^[^ ^28^ ^]^ long non‐coding RNA (lncRNA)TUC339 in hepatocellular carcinoma,^[^ ^29^ ^]^ exosomal proteins, including CD63 and palmitoylated proteins in breast cancer,^[^ ^30^, ^31^ ^]^ phospho‐signal transducer and activator of transcription 3 (STAT3) in glioblastoma,^[^ ^32^ ^]^ cytoskeleton‐centric proteins in colon cancer,^[^ ^33^ ^]^ intercellular cell adhesion molecule‐1, arachidonic acid (AA), and V‐Ki‐ras2 Kirsten rat sarcoma viral oncogene homolog (KRAS)^G12D^ in pancreatic cancer,^[^ ^34^, ^35^ ^]^ and MFG‐E8T protein in prostate cancer are involved in M2‐like polarization of macrophages.^[^ ^36^ ^]^ miRNAs loaded in exosomes including miR‐1246 in glioma,^[^ ^37^ ^]^ miR‐103a in lung cancer,^[^ ^38^ ^]^ miR‐222‐3p, ‐940 in epithelial ovarian cancer (EOC),^[^ ^39^, ^40^ ^]^ miR‐29a‐3p in oral squamous cell carcinoma (OSCC),^[^ ^41^ ^]^ miR‐203, ‐145 in colorectal cancer (CRC),^[^ ^42^, ^43^ ^]^ miR‐155, ‐125b in pancreatic cancer, and miR‐25‐3p, ‐92a‐3p in liposarcoma can drive the polarization of M2 macrophages.^[^ ^44^, ^45^ ^]^ Additionally, miR‐375 is delivered by LDL particles in apoptotic breast cancer,^[^ ^25^ ^]^ miR‐21, ‐511, ‐9, and the extracellular matrix metalloproteinase inducer delivered by microvesicles in CRC can also induce M2‐like polarization of macrophages.^[^ ^46^, ^47^ ^]^ The continuous release of EVs from tumors contributes to the ongoing activities of macrophage hijacking and phenotype transformation, resulting in M2‐like TAMs becoming the primary component of solid tumors^[^ ^48^ ^]^ (Figure 2B,C).

### 2.4 Macrophages deliver malignant feedbacks

#### 2.4.1 Hijacked macrophages prompt tumor growth and metastasis

**FIGURE 3:**
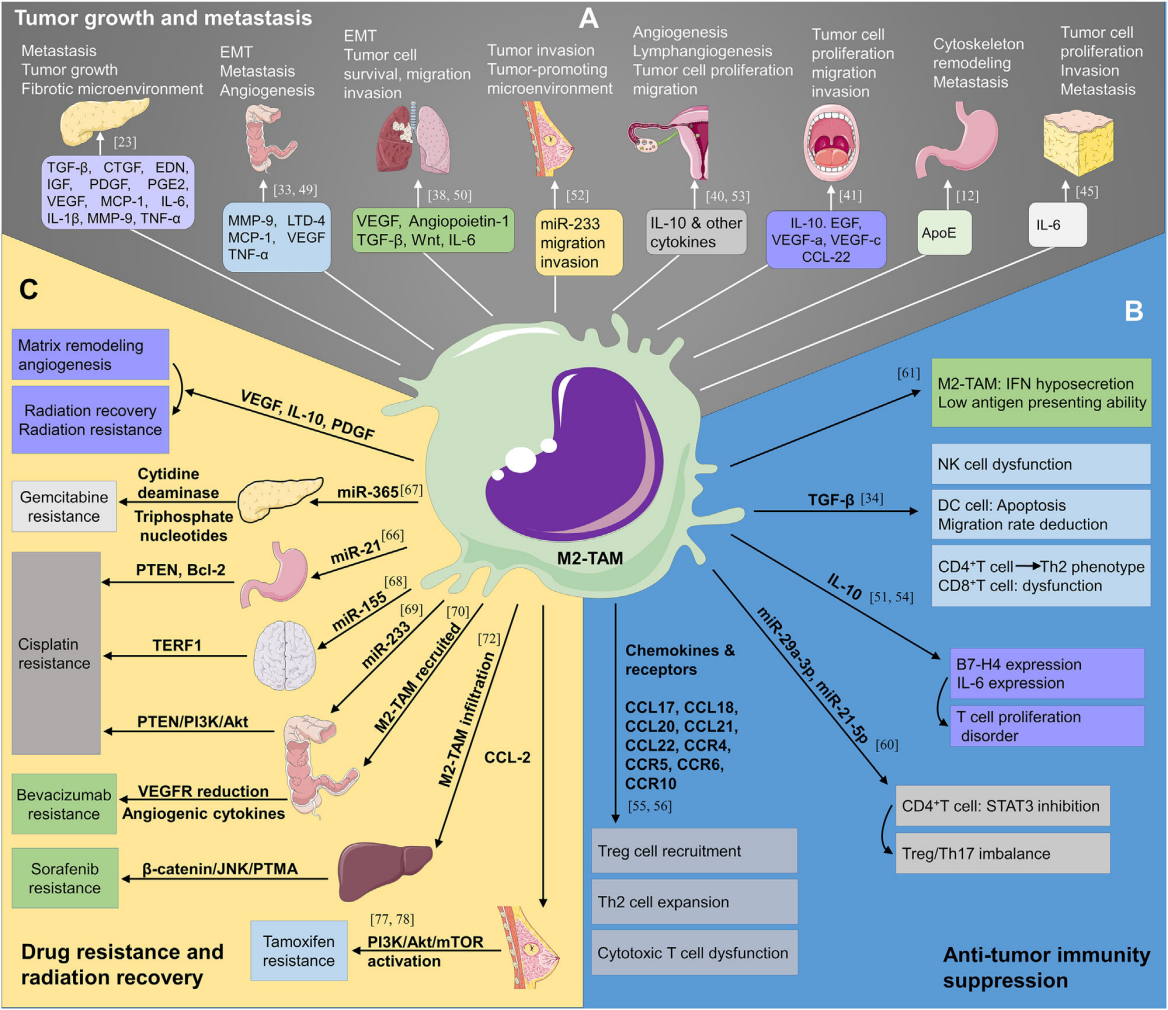
M2‐like TAMs deliver malignant feedback to tumors. (A) The factors derived from M2‐like TAMs like TGF‐β, CTGF, EDN, IGF, PDGF, PGE2, VEGF, MCP‐1, IL‐6, IL‐1β, MMP‐9, and TNF‐α in pancreatic cancer, MMP‐9, LTD‐4, MCP‐1, VEGF, and TNF‐α in colon cancer, VEGF, angiopoietin‐1, TGF‐β, Wnt, and IL‐6 in lung cancer, miR‐233 in breast cancer, IL‐10 in epithelial ovarian cancer, IL‐10, EGF, VEGF‐a, VEGF‐c, and CCL‐22 in oral squamous cell carcinoma, ApoE in gastric cancer, IL‐6 in liposarcoma are involved in the tumor growth and metastasis. (B) M2‐like TAMs can inhibit the tumor‐related immune response. M2‐TAMs‐derived factors like TGF‐β, IL‐10, miR‐29a‐3p, and miR‐21‐5p can participate in the biological processes of NK cell inactivation, DC apoptosis, CD4^+^, and CD8^+^ T cell dysfunction, Treg cell recruitment, which will create an immunosuppressive microenvironment. (C) M2‐like TAMs facilitate drug resistance and radiation recovery of tumor cells. VEGF, IL‐10, PDGF can promote matrix remodeling and angiogenesis, resulting in radiation resistance and recovery. Additionally, miR‐365 in pancreatic cancer can cause Gemcitabine resistance, miR‐21, miR‐155, miR‐233 in gastric cancer, neuroblastoma, and colon cancer can induce Cisplatin resistance, CCL‐2 in breast cancer can boost tamoxifen resistance. Moreover, the recruitment and infiltration of M2‐TAMs can cause Bevacizumab and sorafenib resistance

Macrophages in the TME can generate tumor‐promoting feedbacks. In pancreatic cancer, Kupffer cells begin to secrete more TGF‐β, connective tissue growth factor, eosinophil‐derived neurotoxin, insulin‐like growth factor, and PDGF after taking up the exosomes from the PDAC, which enhances the production of fibronectin and creates a fibrotic microenvironment before tumor metastasis.^[^ ^23^ ^]^ Macrophages treated with PDAC exosomes can also stimulate the secretion of PGE2, VEGF, monocyte chemo‐attractant protein (MCP)‐1, IL‐6, IL‐1β, MMP‐9, and TNF‐α. They have been proven to be related to pancreatic tumor growth and metastasis.^[^ ^34^ ^]^ In CRC, after phagocytosis of the exosomal miR‐145, macrophages are transformed into M2‐like TAMs, and this type of TAMs can promote angiogenesis and tumor growth by producing VEGF expression and tissue infiltration.^[^ ^43^ ^]^ In addition, cytoskeletal rearrangement and increased secretion of TNFα, leukotriene D4, and MCP‐1 can also be seen in macrophages activated by CRC‐derived EVs.^[^ ^33^, ^49^ ^]^ They can also accelerate CRC growth and metastasis through the epithelial‐mesenchymal transition (EMT) due to MMP‐9 expression and activation.^[^ ^49^ ^]^ In lung cancer, miR‐150 from THP‐1 cells (human myeloid leukemia mononuclear cells) and miR‐103a from hypoxic lung cancer cells can stimulate the secretion of VEGF and angiopoietin‐1 in TAMs after internalization, which provides facilities for the survival, migration, and invasion of tumor cells.^[^ ^38^, ^50^ ^]^ Moreover, the defected macrophages can also accelerate the secretion of IL‐6, Wnt, and TGF‐β, which are stimulative for EMT.^[^ ^51^ ^]^ In breast cancer, macrophages can be activated by miR‐375 from apoptotic breast cancer, and M2‐like TAMs have been found to infiltrate tumors, which drives the formation of a tumor‐promoting microenvironment.^[^ ^25^ ^]^ Additionally, the activated TAMs can transport miR‐233 to breast cancer cells and enhance the invasion of cancer cells through the MEF2c‐β‐Catenin pathway.^[^ ^52^ ^]^ In EOC, after being induced by exosomal miR‐940 and miR‐222‐3p, M2‐like TAMs dramatically promote EOC cells' proliferation and migration. Angiogenesis and lymphangiogenesis in the TME are also reinforced in this microenvironment.^[^ ^40^, ^53^ ^]^ In OSCC, TAMs educated by exosomal miR‐29a‐3p can also exhibit the capability to facilitate cell proliferation and migration.^[^ ^41^ ^]^ In gastric cancer, activated TAMs can transport Apolipoprotein E to cancer cells and subsequently promote the metastasis of gastric cancer by affecting cytoskeleton remodeling through the phosphatidylinositol 3‐kinase (PI3K)‐Akt pathway.^[^ ^12^ ^]^ In liposarcoma, the M2‐like TAMs can be activated by exosome‐derived miR‐25‐3p and miR‐92a‐3p, which in turn promote liposarcoma cell proliferation, invasion, and metastasis via a toll‐like receptor (TLR) 7/8‐dependent method^[^ ^45^ ^]^ (Figure 3A).

#### 2.4.2 Hijacked macrophages inhibit the tumor‐related immune response

TAMs activated in the TME can also show the potential of immune regulation, including the inhibition of the viability of T cells and the secretion of chemokines to recruit T cells without cytotoxic capacity. TDEVs can decrease the antigen‐presenting ability of M2‐like TAMs and raise the generation of cytokines such as TGF‐β, IL‐10, and PGE2.^[^ ^34^, ^51^, ^54^ ^]^ TGF‐β mediated by TAMs can not only prevent NK (natural killer) cells from killing tumor cells, promote dendritic cell (DC) apoptosis and reduce its migration rate, but also promote the differentiation of CD4^+^ T cells into the Th2 phenotype, and then inhibit the anti‐tumor activity of CD8^+^ T cells. M2‐like TAMs derived from IL‐10 can upregulate the expression of the B7‐H4 gene alone or together with IL‐6 and then inhibit the activity and proliferation of T cells. TDEVs also induce the release of chemokines (CCL17, CCL18, CCL20, CCL21)^[^ ^55^ ^]^ and receptors (chemokine receptor (CCR)4, CCR5, CCR6, CCR10) by the M2‐like TAMs,^[^ ^56^ ^]^ which are linked to the recruitment and an increase in regulatory T cells (Treg cells) and Th2 cells to the TME, leading to the function impair of cytotoxic T cells.^[^ ^22^, ^55^, ^57^ ^]^ In EOC, macrophage infiltration has been positively correlated with the quantity of Treg cells.^[^ ^58^ ^]^ Additionally, CCL22 secreted by TAMs is involved in Treg regulation by the CL22/CCR4 axis.^[^ ^59^ ^]^ Furthermore, exosomes from activated M2‐like TAMs can transfer miR‐29a‐3p and miR‐21‐5p targeting STAT3 to T cells, resulting in a Treg/Th17 imbalance and tumor immune escape.^[^ ^60^ ^]^ Activated exosomal epidermal growth factor receptor (EGFR) from lung cancer can also be taken up by TAMs, which engage a macrophage‐intrinsic signaling pathway called kinase mitogen‐activated protein kinase kinase 2 (MAP3K8/MEKK2), affecting the production of IFN‐1 and causing immunocompromised host immunity.^[^ ^61^ ^]^ In CRC and breast cancer, the phagocytic and anti‐tumor properties of TAMs with high expressions of programmed cell death protein 1 (PD‐1) are significantly weakened^[^ ^62^ ^]^ (Figure 3B).

#### 2.4.3 Hijacked macrophages contribute to drug resistance and radiation recovery

Although radiotherapy and drug therapy (e.g., chemotherapy and targeted therapy) are still irreplaceable as the current model of cancer therapy,^[^ ^63^ ^]^ many patients still do not benefit from radiotherapy and chemo‐radiotherapy due to resistance.^[^ ^64^, ^65^ ^]^ In the TME, activated M2‐like TAMs can transfer miR‐21 to gastric cancer cells through exosomes and then enhance the anti‐apoptosis ability of cancer cells by regulating the expression level of phosphatase and tensin homolog deleted on chromosome 10 (PTEN) and B‐cell lymphoma‐2 (Bcl‐2) and reinforce the resistance of gastric cancer cells to Cisplatin.^[^ ^66^ ^]^ In PDAC, exosomal miR‐365 derived from infiltrating macrophages can be internalized into tumor cells, disrupting the activity of Gemcitabine by inducing the expression of cytidine deaminase and upregulating the level of triphosphate nucleotides in cancer cells.^[^ ^67^ ^]^ In neuroblastoma, the internalized miR‐155 from TAMs can target the signaling pathway of telomeric repeat binding factor 1, thus increasing the resistance of neuroblastoma cells to chemotherapy drugs.^[^ ^68^ ^]^ Additionally, miR‐223 from M2‐like TAMs can be taken up by EOC cells and assist tumor cells in resisting anti‐tumor drugs' effects by targeting the PTEN/PI3K/AKT pathway.^[^ ^69^ ^]^ Activated TAMs are also involved in targeted cancer therapy. In EOC, macrophages are also responsible for the induced bevacizumab resistance owing to the low expression of vascular endothelial growth factor receptor and excessive secretion of angiogenic cytokines.^[^ ^70^ ^]^ In gastrointestinal stromal tumors, the signaling pathway of C/EBP may be abnormally activated after imatinib treatment, leading to the transformation of M1‐like TAMs to tumor‐promoting M2‐like TAMs.^[^ ^71^ ^]^ The defected macrophages can also resist targeted therapy (sorafenib) by activating the β‐catenin/c‐JNK/PTMA pathway.^[^ ^72^ ^]^ Myeloid lymphocytes proliferation and the release of inflammatory cytokines can be caused by radiation, resulting in M2‐like polarization,^[^ ^19^, ^23^, ^73^, ^74^, ^75^ ^]^ EMT,^[^ ^51^ ^]^ immunosuppression,^[^ ^34^, ^51^, ^54^ ^]^ and tumor metastasis.^[^ ^23^ ^]^ The M2‐like TAMs caused by radiation can promote the repair of damaged tumor tissue using matrix remodeling and neo‐angiogenesis.^[^ ^76^, ^77^ ^]^ Moreover, in endocrine therapy, M2‐like TAMs can stimulate the PI3K/Akt/mammalian target of the rapamycin (mTOR) signaling pathway and produce endocrine resistance feedback.^[^ ^77^, ^78^ ^]^ Finally, in immunotherapy, Treg cells and TAMs can serve the weakening of anti‐tumor immunity through the depressing of T cells and secretion of inhibitory factors^[^ ^79^, ^80^ ^]^ (Figure 3C).

## 3 TUMORS AND MICROBIOTA

### 3.1 Tumors recruit microbiota through extracellular vehicles

**FIGURE 4:**
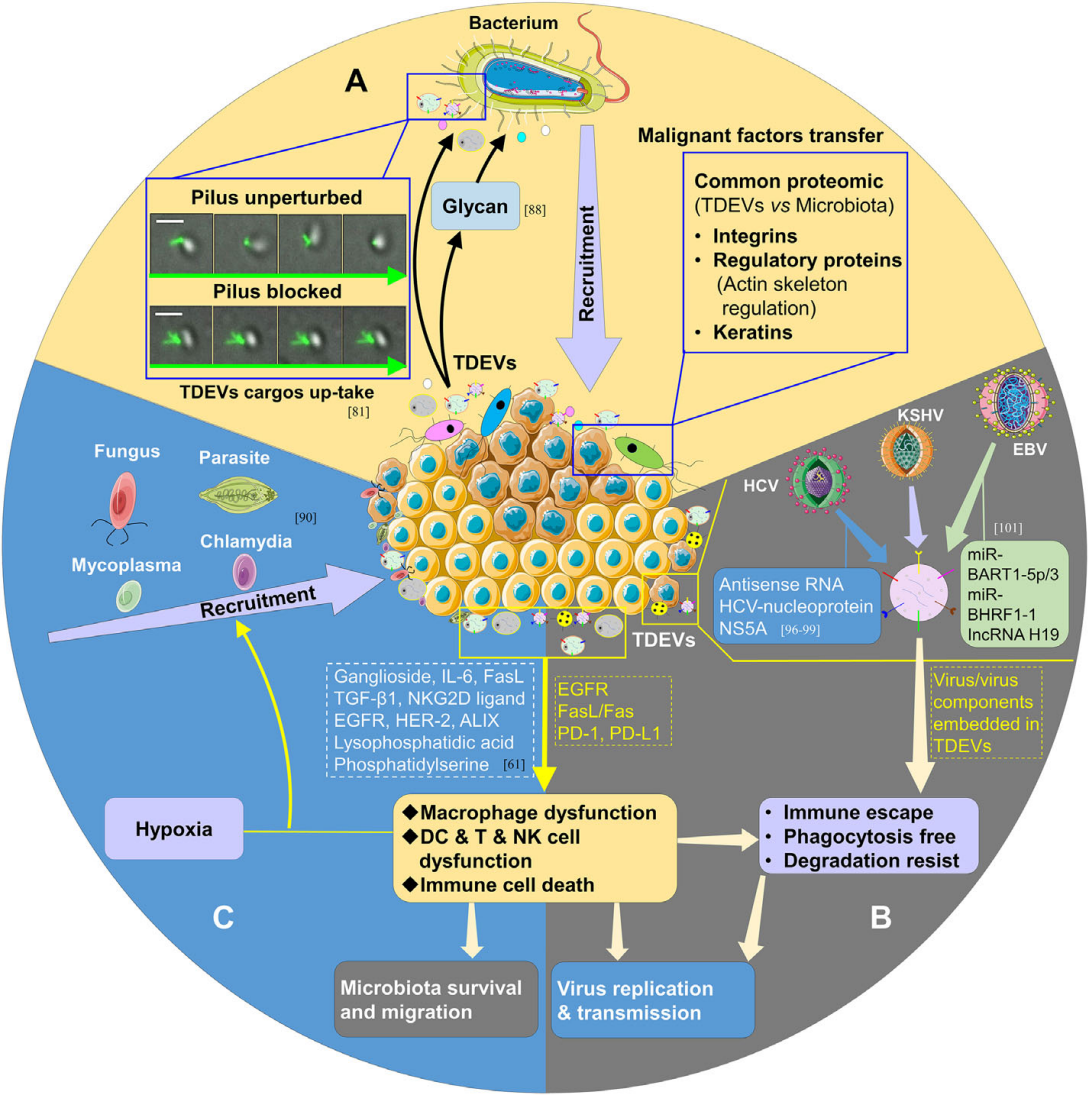
Tumor cells recruit and foster the survival and migration of microbiota via TDEVs. (A) Tumor cells recruit and deliver malignant factors to bacteria. In CRC, tumor cells can recruit some tumor‐related bacteria like *Fusobacterium* by releasing glycan. Additionally, they can transfer integrins, regulatory proteins, and keratins to the recruited microbiota. Images adapted with permission.^[^ ^81^ ^]^ Copyright 2017, The American Association for the Advancement of Science. (B) Tumor cells promote replication and transmission of the virus. The tumor‐derived factors like EGFR, Fasl/Fas, PD‐1, PD‐L1 can foster the dysfunction of macrophages, DC, T cells, and NK cells, and TDEVs can also help microbiota transfer malignant factors, which will accelerate the replication and transmission of microbiota. (C) Tumors guarantee the survival and migration of microbiota. Similarly, the tumor‐derived factors like ganglioside, IL ‐6, FasL, TGF‐β1, NKG2D ligand, EGFR, HER‐2, and ALIX can induce the dysfunction of macrophages, DC, T cells, and NK cells, combined with hypoxia; the microbiota can be recruited into the tumor microenvironment and have long‐term survival

The horizontal transfer of genes occurs when exogenous genes are introduced into host cells.^[^ ^81^ ^]^ In addition, exosomal miRNA and other EVs derived from plants can shape the gut microbiota and fungi. After being preferentially taken up in the gut, the contents in EVs can cause structure and localization of bacteria and fungi, resulting in significant morphological changes and anti‐inflammatory effects.^[^ ^82^, ^83^ ^]^ Furthermore, it has been certified that miRNA from host feces can achieve the gene regulation of gut microbiota.^[^ ^84^ ^]^ In the TME, tumor cells can secret more aggressive EVs than nonmalignant ones.^[^ ^85^ ^]^ Some TDEVs are acknowledged as communication mediators between tumors and microbiota.^[^ ^86^, ^87^ ^]^ Most notable is the interaction between TDEVs and bacteria.^[^ ^2^ ^]^ The overproduction of glycan from CRC cells can raise the migration of tumor‐related bacteria like *Fusobacterium* to the TME.^[^ ^88^ ^]^ EVs from CRC can modulate bacteria in the gut since they have been found to share some common proteomics,^[^ ^7^ ^]^ and the signaling pathway in which the matching sequence participates is notably associated with poor outcomes of CRC.^[^ ^89^ ^]^ This means CRC can transfer malignant factors to bacteria through EVs. Until now, the mechanisms by which EVs enter bacteria and interfere with specific target genes have remained unclear.^[^ ^40^ ^]^ Reports state that EVs and their cargos may enter bacteria by endocytosis or through surface sensing, extension, and pilus retraction activity.^[^ ^81^ ^]^ Moreover, EVs‐loaded hypoxia factors in the TME can also help recruit mycoplasma and chlamydia^[^ ^90^ ^]^ (Figure 4A).

### 3.2 Tumors shield microbiota through Extracellular vehicles

Viruses like *papillomavirus* (HPV), *hepatitis B virus* (HBV), *Epstein‐Barr virus* (EBV), and *Kaposi's sarcoma herpesvirus* (KSHV) can lead to cervical carcinoma, hepatocellular carcinoma, nasopharyngeal carcinoma, and Kaposi's sarcoma to some degree, respectively.^[^ ^91^, ^92^, ^93^, ^94^ ^]^ Large amounts of experiments have confirmed that TDEVs may be involved in the structure or function of various immune cells. Briefly, TDEVs can drive the apoptosis of CD8^+^ T cells and inhibit their proliferation; they can also reduce the cytotoxicity of NK cells and boost the proliferation of Tregs and myeloid‐derived suppressor cells (MDSCs). In another study, macrophages can internalize EGFR‐loaded TDEVs, targeting MEKK2 and interferon regulatory factor 3 (IRF3), which substantially suppressed the production of IFN‐βand increased the susceptibility to viruses.^[^ ^61^ ^]^ Generally, after invading the host, the microbiota may be eliminated by macrophages, DC, T cells, B cells, and NK cells. An immunosuppressive niche built by TDEVs in the TME may help microbiota escape from immune injury. In these circumstances, the microbiota may remain alive or aggressive under the cover of barriers from TDEVs.

### 3.3 Tumors support replication and transmission of microbiota through extracellular vehicles

Beyond that, TDEVs also play a vital role in virus replication and transmission.^[^ ^95^ ^]^ Exosomes from hepatitis C virus (HCV)‐associated HCC can carry genetic information from HCV,^[^ ^96^ ^]^ including antisense RNA with replication potential,^[^ ^97^ ^]^ HCV nucleoprotein, and non‐structural protein 5A (NS5A).^[^ ^98^, ^99^ ^]^ When transferred to susceptible cells, HCV can expand its domination.^[^ ^100^ ^]^ In EBV‐positive lymphoma, the exosomes secreted by tumor cells contain EBV‐derived miRNAs (e.g., EBV‐miRNA‐BART1‐5p, EBV‐miR‐BART3, and EBV‐miR‐BHRF1‐1) and IncRNAs (e.g., IncRNA H19).^[^ ^101^ ^]^ Thereafter, the EBV and its unique factors may be transferred to uninfected recipient cells and exert hazardous action.^[^ ^102^ ^]^ In Kaposi's sarcoma, KSHV can be embodied in EVs from tumor cells; the lipid bilayer structure is the fundamental constitution of EVs that can protect cargos (e.g., DNAs and RNAs) from degradation.^[^ ^10^, ^103^ ^]^ These EVs will ultimately carry KSHV to uninfected cells and develop an egoistic microenvironment for viral replication and transmission^[^ ^104^ ^]^ (Figure 4B,C).

### 3.4 Microbiota facilitates tumor progression

#### 3.4.1 Microbiota induces tumor‐associated inflammatory environment

**FIGURE 5:**
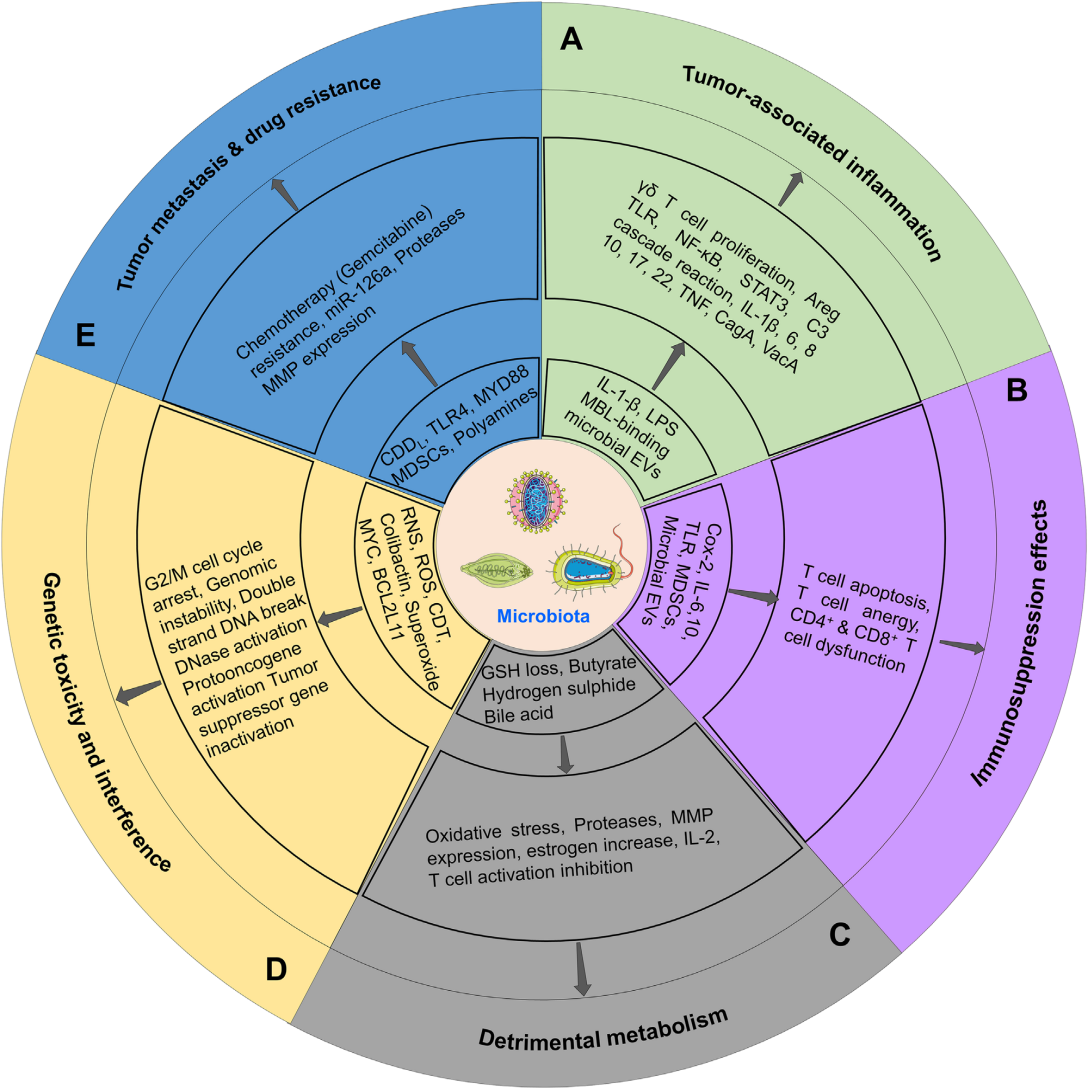
The microbiota transmits malignant feedback to tumors. (A) The microbiota induces a tumor‐associated inflammatory environment. The microbiota in the tumor microenvironment can activate the signaling pathways of NF‐κB/MAPK, TLR, STAT3, and C3 cascade reaction by boosting the release of IL‐1β, IL‐6, IL‐8, IL‐10, IL‐17, IL‐22, IL‐23, and LPS. (B) The microbiota generates immunosuppressive effects in the tumor microenvironment. In the tumor microenvironment, the microbiota can cause T cell dysfunction and innate immune repression by generating factors like COX‐2, IL‐6, IL‐10, TLR ligand, MDSCs, and EVs. (C) The microbiota produces detrimental metabolism in the tumor microenvironment. In microbiota‐related metabolism, iron metabolic disorder, GSH can cause oxidative stress, the production of polyamines can induce T cell inhibition, and the expression of proteases and MMP, the generation of β‐glucuronidase can impede the inactivation of estrogens, consequently increasing the level of estrogen and stimulating the growth of estrogen^+^ tumors. (D) The microbiota promotes tumor progression through genetic toxicity and interference. The productions from microbiota like RNS, ROS, CDT, colibactin, superoxide are inductive factors for DNA strand damage, cycle arrest, point mutation, and gene reprogram. (E) The microbiota promotes tumor progression by contributing to metastasis and drug resistance. Autophagy pathway activation, TLR4, and MYD88 pathway activation can lead to Gemcitabine resistance; further, the generation of polyamines and MDSCs can stimulate the tumor metastasis

The intratumor microbiotas never live together peacefully and may discriminate against each other,^[^ ^105^ ^]^ with some tumor‐promoting microbiotas establishing dominance with the help (directly or indirectly) of TDEVs. Subsequently, these microbiotas will evoke more tumor‐associated inflammation. The major bacterial community of lung adenocarcinoma can enhance the release of pro‐inflammatory factors, which will generate the harmful inflammatory response and malignant proliferation of cells.^[^ ^6^ ^]^ IL‐17 and IL‐23 secretions are observed in familial adenomatous polyposis and CRC, closely related to inflammation and carcinogenesis.^[^ ^106^ ^]^ In other gastrointestinal tumors, some types of bacteria (*Klebsiella* and *Haemophilus*) are significantly increased and active in lipopolysaccharide (LPS) production.^[^ ^107^ ^]^ The interaction between LPS and TLRs can stimulate the expression of pro‐inflammatory cytokines, such as NF‐κB.^[^ ^108^, ^109^, ^110^ ^]^ Furthermore, LPS‐TLR can activate the STAT3‐related pathways and tumor progression by triggering the KRAS mutation.^[^ ^111^, ^112^ ^]^ In pancreatic cancer, *Malassezia* spp. can migrate to the pancreas and activate mannose‐binding lectin (MBL), a kind of protein, which can recognize and bind to glycans of the fungal wall and trigger cascade reactions of the complement system (C3). Later, MBL‐C3 induces inflammation and oncogenic progression.^[^ ^113^ ^]^ Apart from these mediators, EVs derived from microbiota can also accelerate the formation of a tumor‐associated inflammatory environment. They boost tumor‐associated inflammation by either carrying/inducing IL‐6, IL‐8, IL‐10, and TNF or participating in the inflammatory process as they promote recruitment and infiltration of inflammatory cells.^[^ ^114^ ^]^ For instance, *H. pylori* can induce the expression of IL‐1β, IL‐6, IL‐8, and IL‐17 through cytotoxin‐associated gene product A (CagA) and vacuolating cytotoxin gene A (VacA) in EVs.^[^ ^115^ ^]^ Generally, carcinogenic microbiotas survive, replicate, and become dominant in the tumor, and they take up malignant characteristics from the tumor and even migrate with tumor cells.^[^ ^116^ ^]^ In turn, they initiate tumor‐related inflammation, which serves the progression of the tumor (Figure 5A).

#### 3.4.2 Microbiota produces immunosuppressive effects

In the immune‐disordered microenvironment, microbiomes themselves can also participate in immunosuppressive activities. EVs secreted by *H. pylori* can increase cyclooxygenase‐2 and restrain T cell responses.^[^ ^117^ ^]^ In addition, EVs derived from *H. pylori* can also increase the expression of immunosuppressive cytokines like IL‐6 and IL‐10 and lead to the apoptosis of T cells.^[^ ^118^ ^]^ In PDAC, some microbiome suppressed the efficacy of checkpoint‐based immunotherapy by disturbing immune tolerance.^[^ ^119^ ^]^ In the PD‐1 immunotherapy of melanoma, the low diversity and high abundance of *Bacteroidales* have been demonstrated to be limited in the capacity of antigen presentation and deleterious for systemic and anti‐tumor immune responses.^[^ ^120^ ^]^ In some opportunistic fungal pathogens, *C. tropicalis* has been proved to accelerate the transformation of myeloid cells to MDSCs. MDSCs can significantly inhibit CD4^+^ and CD8^+^ T cell responses and drive tumor progression.^[^ ^121^ ^]^ Additionally, innate immunity may be compromised when parasites invade the hosts. Some secreted nematode (*H. polygyru*s) miRNAs delivered by exosomes could suppress type II innate responses after being internalized by host cells.^[^ ^122^ ^]^ *N. caninum* EVs could interact with TLR‐2 and activate the mitogen‐activated protein kinase (MAPK) signaling pathway, thus repressing the host's innate immune responses.^[^ ^123^ ^]^ In addition, the MAPK signaling pathway may also play a vital role in both inflammation and tumorigenesis.^[^ ^109^, ^110^ ^]^ During the chronic stage of infection, the EVs‐enclosed miRNAs from *Schistosoma* can tune down the T cell‐related immune responses, which account for both the long‐term existence of *Schistosoma* and *Schistosoma*‐related tumor progression.^[^ ^124^ ^]^ Researchers have also confirmed that EVs of *F. hepatica* are enriched in some potential immune‐regulatory proteins and miRNAs.^[^ ^125^, ^126^ ^]^ These factors work together to ensure the survival of *F. hepatica* through host immunosuppression^[^ ^126^ ^]^ (Figure 5B).

#### 3.4.3 Microbiota causes detrimental metabolism

In the human gut, commensal microbiomes are involved in the absorption and digestion of nutrients (sugar, fat, amino acid),^[^ ^127^ ^]^ metabolism of hormones and bile acids, synthesis of vitamins (e.g., vitamin B and vitamin K),^[^ ^128^, ^129^ ^]^ and the formation of intestinal physical (mucosal) and chemical (immune) barriers.^[^ ^130^, ^131^, ^132^ ^]^ However, in TME, the education of the TDEVs brings about tremendous diversion in the composition and abundance of the microbiota. What follows is some harmful metabolism, which may stimulate tumor deterioration. Microbiota can participate in host metabolic activities. Also, they can give tumors feedback with toxic and harmful metabolites.^[^ ^131^, ^133^, ^134^, ^135^ ^]^ Apart from LPS in tumor‐associated inflammation,^[^ ^107^ ^]^ some other harmful metabolism processes should also be noted. It was found that the toxigenic *H. pylori* strain can induce the formation of micronuclei and vacuolation in normal gastric epithelial cells. This is followed by iron metabolism disorder and glutathione (GSH) loss, prompting a role for oxidative stress in carcinogenesis.^[^ ^136^ ^]^ Polyamines and the production of some gut bacteria can also be produced by host cells under the stimulation of certain gut bacteria (e.g., enterotoxigenic *Bacteroides fragilis*),^[^ ^137^, ^138^ ^]^ which have been proven to be active in suppressing anti‐tumor immunity via the inhibition of IL‐2 and activated T cells.^[^ ^139^ ^]^ They can also raise the expression of proteases and matrix metalloproteinases in the tumor, resulting in an enhanced invasion and migration of tumor cells.^[^ ^140^ ^]^ The microbiota also becomes involved in the hormone metabolism of the host.^[^ ^90^ ^]^ Especially in hormone‐receptor‐positive breast cancer individuals, β‐glucuronidase, a kind of bacterial enzyme that can impede the binding of glucuronic acid and estrogens, increases the level of estrogen in the host and promotes tumor growth.^[^ ^141^, ^142^ ^]^ In a recent study of CRC, the microbiota‐derived metabolite butyrate was shown to be effective in the hyperproliferation of intestinal epithelial cells and the development of CRC.^[^ ^143^ ^]^ Some other studies have also confirmed the detrimental role of hydrogen sulfide, bile acid, and ethanol metabolism, which could be driven by commensal microbiota^[^ ^133^ ^]^ (Figure 5C).

#### 3.4.4 Microbiota brings genetic toxicity and interference

Genetic toxicity and genetic interference are accumulated through microbiota feedback. Typically, ROS, reactive nitrogen species, and other certain toxins are mediated by the microbiota, which can induce DNA damage.^[^ ^144^, ^145^ ^]^ Cytolethal distending toxin is a production of gram‐negative bacteria (e.g., *E. coli*, *Helicobacter* spp., and *S. typhi*) and can be carried by *C. jejuni* and *Helicobacter cinaedi*,^[^ ^146^ ^]^ which is associated with gastric, colorectal, and gallbladder cancer progression.^[^ ^147^ ^]^ With this infection, this kind of toxin may be released, and one of its subunits, called cytolethal distending toxin B, can be transferred into the nucleus of host cells, exerting DNA damage mediated by DNase activity, as well as cell swelling and transient G2/M cell cycle arrest. The potential mechanisms may include the activation of the histone H2AX phosphorylation, ataxia‐telangiectasia mutated‐cell cycle checkpoint kinase 2, and the mTOR signaling pathway.^[^ ^145^, ^148^ ^]^ Another bacterial toxin called colibactin derived from certain *E. coli* strains can alkylate DNA with an unusual electrophilic cyclopropane, generating an unstable colibactin‐DNA adduct, leading to severe DNA damage and CRC progression.^[^ ^149^, ^150^, ^151^ ^]^ Moreover, some other metabolites (e.g., superoxide radicals and hydrogen sulfide) from microbiota can also elicit genetic toxicity. For instance, large quantities of superoxide can be produced by *Enterococcus faecalis*, which will bring about double‐strand DNA damage and chromosome instability.^[^ ^152^ ^]^ In EBV‐associated lymphoma, the virus can reprogram the genes of MYC and BCL2L11 and form a new DNA loop in which the MYC gene has closer contact with its enhancer, while BCL2L11 is blocked to contact its enhancer. As a result, the proliferation of tumor cells is enhanced with apoptosis inhibited.^[^ ^153^ ^]^ In CRC, several matching microbial sequences have been found in the colorectal EV proteome, which means the microbiota can transfer their genetic fragments to host cells.^[^ ^7^ ^]^ In the viral field, insertional carcinogenesis has been universally recognized as a core process of genetic interference. While recent studies also elaborate that bacterial and parasitic DNA may also integrate into the host genome and become oncogenic. This type of lateral gene transfer may profoundly contribute to tumor progression^[^ ^154^ ^]^ (Figure 5D).

#### 3.4.5 Microbiota fosters tumor metastasis and drug resistance

Apart from the above feedback mechanisms from the microbiota to tumors, specific microbiota is also involved in the metastasis and drug resistance during tumor progression. As has been noted, some bacteria can produce polyamines,^[^ ^137^ ^]^ and in the TME, tumor cells can increase the uptake of polyamines from certain bacteria. Resulting in MMP production and further acceleration of tumor spread.^[^ ^140^ ^]^ In addition, some colonizing microbes in CRC have been found maintained in distal metastases, illuminating the fact that the microbiota can metastasize with tumor cells and form a TME like the primary sites.^[^ ^116^ ^]^ As mentioned above, *C. tropicalis* can promote the formation of MDSCs; furthermore, miR‐126a delivered by MDSC exosomes has been confirmed to be active in promoting lung metastasis.^[^ ^155^ ^]^ Specific microbiota can also engender the resistance of anti‐tumor therapy. In CRC, the autophagy pathway activation and the chemotherapeutic response inhibition are induced by *F. nucleatum*, which activates TLR4, and myeloid differentiation factor (MYD)88 signaling pathway and specifically downregulates the synthesis of miR‐18a and miR‐4802 to assist cancer cells in resisting chemotherapy.^[^ ^156^ ^]^ The production of cytidine deaminase (CDD_L_) from gammaproteobacterial was also responsible for gemcitabine inactivation in PDAC.^[^ ^157^ ^]^ Dramatically, gemcitabine lost effectiveness in breast cancer‐bearing *M. hyorhinis*.^[^ ^158^ ^]^ Tumor metastasis and drug resistance are nonnegligible factors that account for the refractoriness of some tumors. In this microenvironment with complex interplay, more mechanisms remain to be revealed (Figure 5E).

## 4 MACROPHAGES AND MICROBIOTA

### 4.1 Macrophages indulge microbiota

#### 4.1.1 Macrophages present impaired phagocytic capability

**FIGURE 6:**
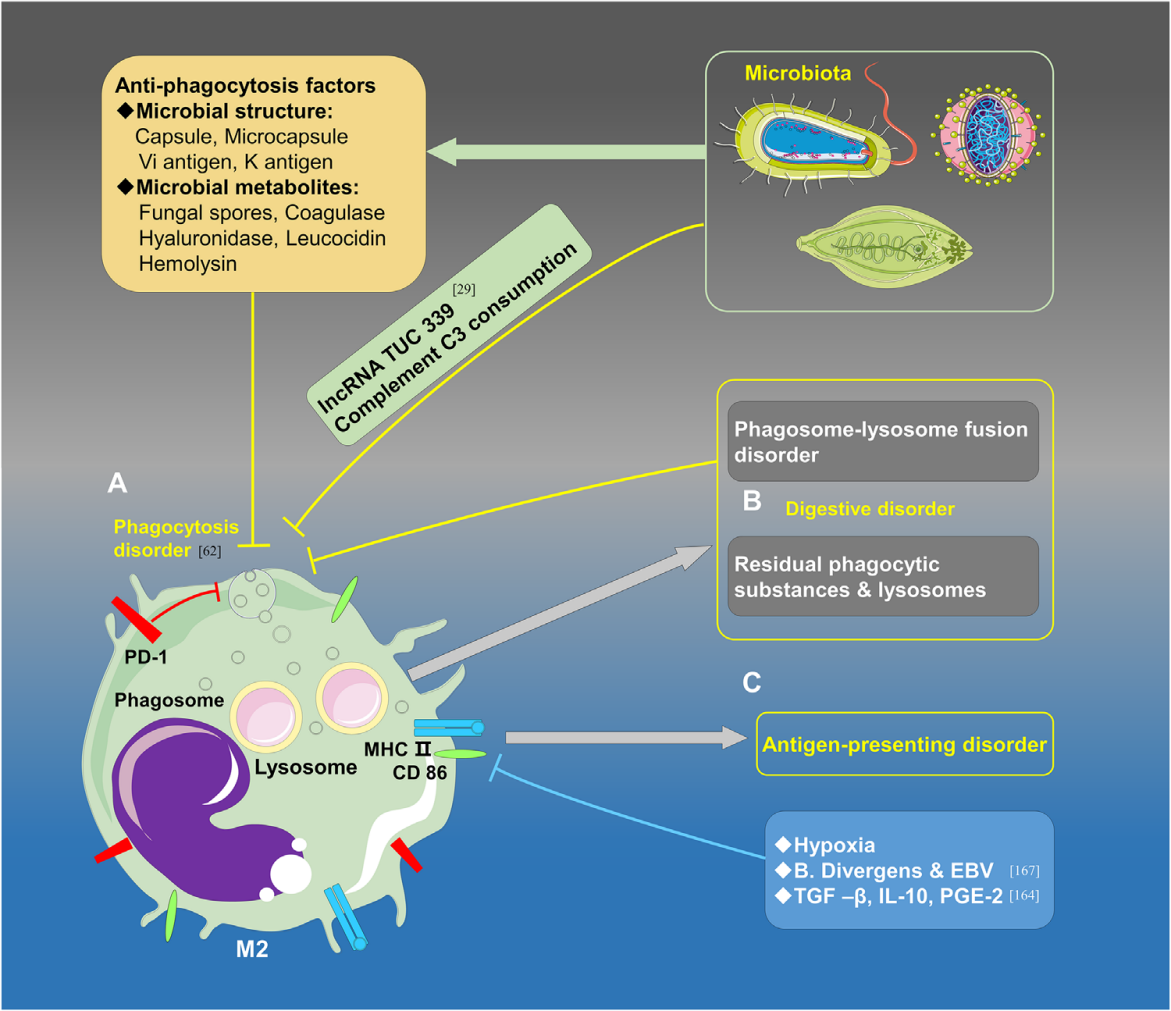
M2‐like TAMs indulge the survival of the microbiota in the tumor microenvironment. (A) The phagocytic capacity of M2‐like TAMs is weakened under the stimulation of endogenous factors and anti‐phagocytosis factors derived from the microbiota. PD‐1, lncRNA TUC 339, and complement C3 consumption along with some microbial structures (capsule, microcapsule, Vi antigen, K antigen), microbial metabolites (fungal spores, coagulase, etc., hyaluronidase, leucocidin, hemolysin) may lead to the phagocytosis disorder of the macrophages. (B) The digestive capacity of M2‐like TAMs is abated due to phagosome‐lysosome fusion disorder and extensive intracellular residues. (C) The antigen‐presenting capacity of M2‐like TAMs is diminished due to the infection of B. Divergens and EBV and the inhibitory effects of hypoxia, TGF‐β, IL‐10, and PEG‐2 on the expressions MHC II and CD86

Unfortunately, macrophages do not work as the first barrier of human innate immunity to phagocytize invaders after being hijacked by TDEVs.^[^ ^2^ ^]^ TAMs in solid tumors mainly present the M2 phenotype, which contributes to the formation of the immunosuppressive microenvironment.^[^ ^159^ ^]^ M2 is inferior to M1 in the ability of tumor clearance, especially after being hijacked by TDEVs. In HCC, the M2 phenotype polarization is stimulated by the exosomal lncRNA TUC339 derived from cancer cells, which weakens phagocytosis in macrophages and reduces pro‐inflammatory cytokine secretion in TME.^[^ ^29^ ^]^ Moreover, a research group also provided insight into the PD‐1 expression of TAMs. They observed that whether in human or mouse cancer models, the PD‐1 expression of TAMs can increase with time or throughout the disease. The expression of PD‐1 in TAMs is negatively correlated with the phagocytic capacity of tumor cells. Blocking PD‐1/PD‐L1 in vivo can increase phagocytosis of macrophages, which will decelerate tumor growth and prolong the survival time of the mouse tumor model.^[^ ^62^ ^]^ In addition to common cytokines such as IL‐4, IL‐10, IL‐13, CSF‐1, and TGF‐β, fungal and helminth infections also drive the process of M2‐like TAMs polarization.^[^ ^18^ ^]^ *Schistosoma* infection is closely related to HCC, CRC, and bladder cancer. A new study showed that *Schistosoma japonicum* derived miR‐125b carried by EVs can be taken up by macrophages, which will increase the mRNA expression of TLR signaling pathway‐related molecules. In addition, this will promote the proliferation of macrophages. In addition, the host macrophage functions are regulated to guarantee parasitism.^[^ ^160^ ^]^ Moreover, microorganisms have some mechanisms to protect them from being eliminated by macrophages, such as the capsule, microcapsule, Vi antigen, K antigen, fungal spores, coagulase, hyaluronidase, leucocidin, and hemolysin. Complements play an essential role in mediating the phagocytosis of macrophages; certain microbiota can consume complements (e.g., C3) and suppress phagocytosis by generating some by‐products to activate complements but prevent the formation of antimicrobial effects (Figure 6A).

#### 4.1.2 Macrophages exhibit digestion and antigen presentation disorders

Recognition and phagocytosis are important functions of macrophages, while digestion and antigen presentation play irreplaceable roles in pathogen elimination. It has been shown that apart from tumor cells, macrophages can also express PD‐1/PD‐L1. Over a decade ago, researchers found that macrophages can express a significantly higher level of PD‐1 under an infectious condition, and the PD‐1^+^ macrophages exhibit worse bacterial clearance than PD‐1^−/−^ macrophages.^[^ ^161^ ^]^ In another study, researchers revealed that nearly all PD‐1^+^ macrophages have the characteristics of M2‐like macrophages. Accompanied by tumor progression, PD‐1^+^ M2‐like macrophages not only expanded in quantity but also changed in morphology. An immunohistochemistry test showed that PD‐1^+^ TAMs became swollen and foamy, and electron microscopy illustrated that the foamy appearance resulted from the accumulation of extensive residual phagocytic substances and lysosomes in the cytoplasm.^[^ ^62^ ^]^ In the TME, the digestive capacity of macrophages is weak. For example, in isolated alveolar macrophages, the phagosome‐lysosome fusion is significantly reduced within patients infected with HIV, and digestion‐related organelles are dysfunctional.^[^ ^162^ ^]^ In addition, the secretions of TGF‐β, IL‐10, and PGE2 can down‐regulate the expression of major histocompatibility complex II (MHC‐II) and CD86 in M2‐TAMs, which will suppress the antigen presentation ability. Hypoxia is a common phenomenon in the TME, and some research data have also stated that hypoxia can suppress MHC‐II expression via down‐regulation of the forkhead box O1 in TAMs.^[^ ^163^ ^]^ The specificity of the tumor environment and intrinsic properties of TAMs determine the low antigen‐presenting function of TAMs, triggering a series of exemptions in the microbiota. However, certain microbiota themselves can also regulate antigen‐presenting abilities.^[^ ^164^ ^]^ *Mycobacterium tuberculosis* (MTB) can inhibit the expression of MHC II and CD86 in macrophages induced by IFN‐γ in a dose‐dependent manner.^[^ ^165^ ^]^ The expression of antigen present mediators, MHC‐II or CD86, can be suppressed by cytokines, hypoxia, parasites, and virus.^[^ ^166^ ^]^ Overall, the microbiota can survive and reproduce in the TME benefited from the decline of phagocytosis, digestion, and antigen‐presenting functions (Figure 6B,C).

### 4.2 Microbiota impacts macrophages

#### 4.2.1 Microbiota promotes the phenotypic transformation of macrophages

**FIGURE 7:**
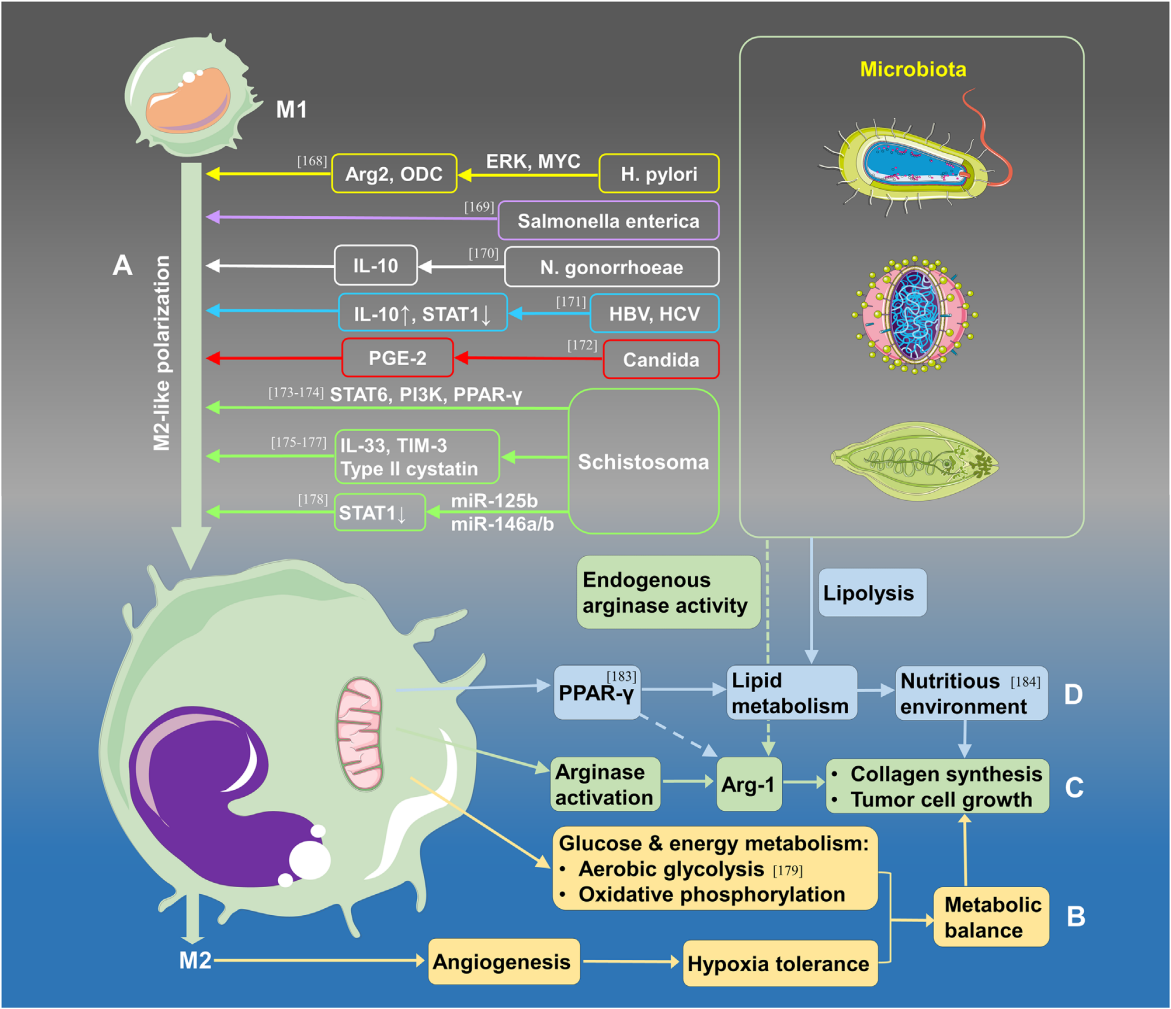
The microbiota accelerates the phenotype and metabolic changes of M2‐like TAMs. (A) The microbiota promotes the phenotype transformation to M2‐TAMs via certain types of cytokines and signaling pathways. The infection of *H. pylori*, *Salmonella enterica*, *N. gonorrhoeae*, HBV, HCV, *Candida*, and *Schistosoma* can promote the formation of M2‐TAMs by affecting the expression of Arg2, ODC, IL‐10, STAT, PGE‐2, IL‐33, TIM‐3, and so on. (B–D) The microbiota induces metabolic alterations of glucose (B), amino acids (C), and lipids (D) in the tumor microenvironment

The microorganisms in TME can complete their life cycle and affect the polarization of M2 in turn. In *H. pylori* infection, some studies have shown that Arginase‐2 (Arg2) plays a vital role in promoting the M2‐like polarization of macrophages. *H. pylori* can also upregulate the level of Ornithine decarboxylase via the extracellular regulated protein kinases and the MYC signaling pathways, which have the same effect as Arg2.^[^ ^167^ ^]^ *Salmonella enterica* can escape damage in humans and mice and proliferate in macrophages by promoting the polarization of the M2 phenotype.^[^ ^168^ ^]^ *N. gonorrhoeae* can raise the level of M2‐like macrophages by stimulating IL‐10 expression.^[^ ^169^ ^]^ Additionally, HBV and HCV are also involved in M2 polarization via the up‐regulation of IL‐10 and the inhibition of STAT1.^[^ ^170^ ^]^ Fungi and parasitic infections can also stimulate the M2‐like phenotypic polarization. For example, PGE2 as a by‐product of Candida can induce M2‐like phenotypic polarization.^[^ ^171^ ^]^ *Schistosoma* can activate STAT6, PI3K signaling pathway and increase M2 polarization.^[^ ^172^, ^173^ ^]^ *Haematobium*, *Clonorchis sinensis*, and *Opisthorchis viverrine* cause the promotion of IL‐33,^[^ ^174^ ^]^ T cell immunoglobulin, mucin domain‐3 (TIM‐3),^[^ ^175^ ^]^ and type II cystatin expression,^[^ ^176^ ^]^ and the inhibition of STAT1 via miR‐146a/b, association with HCC, CRC, and bladder cancer closely.^[^ ^177^ ^]^ Moreover, when the microbial burden in the organism is eliminated, macrophages show the inclination of M1 polarization with a significant increase of CD86, MHC‐II, TNFα, and IL6. This also indirectly proves the relationship between the microbiome and M2.^[^ ^119^ ^]^ During microbial infection, M2 polarization is like a double‐edged sword. M2‐like macrophages can protect the host by clearing microorganisms, suppressing granuloma, repairing tissue damage caused by microbial migration, and limiting the early cytotoxic immune responses. However, M2‐TAMs in TME can also promote tumor progression by enhancing the recruitment and infiltration of pro‐inflammatory macrophages (Figure 7A).

#### 4.2.2 Microbiota induces the metabolic alteration of macrophages

Apart from polarization promotion, metabolic changes also facilitated the malignant progression of tumors. Regulations and feedbacks of metabolism between microbes and macrophages maintain the energy balance in the TME. Although microbial invasion and TDEVs secretion can promote M2‐like polarization, they may compete with tumor cells for energy via oxidative phosphorylation and aerobic glycolysis.^[^ ^178^ ^]^ Angiogenesis is enhanced by M2 polarization leading to high hypoxia tolerance of tumor cells. Furthermore, some parasites, like *Leishmania donovani*, compete with macrophages and other host cells for metabolic nutrients and energy for survival and replication.^[^ ^179^ ^]^ Arginase activity in macrophages may be induced by the microbiota in amino acid metabolism, thus increasing Arg‐1 production; excessive Arg‐1 can accelerate the synthesis of ornithine and then prompt collagen synthesis and tumor cell growth. Endogenous arginase activity in specific parasites may also help raise the level of Arg‐1 in the TME.^[^ ^180^ ^]^

On the other hand, Arg‐1 seems closely related to lipid absorption and oxidative metabolism.^[^ ^181^ ^]^ Some microbes like *Mycobacterium tuberculosis* can escape from immune attack by living inside macrophages via peroxisome proliferator‐activated receptor‐γ (PPARs) pathways and PPAR‐γ expression. Meanwhile, the PPAR‐γ can also participate in Arg‐1 synthesis, M2‐like polarization, and lipid metabolism of the host cells and provide nutrients for the commensal microbes.^[^ ^182^ ^]^ By activating the STAT6‐PPAR‐γ/δ signaling pathway, the polarization of M1‐like macrophages may be inhibited by Some chronic infections (e.g., *Brucella*, *Toxoplasma gondii*), which produce nutrition through lipid oxidation metabolism for pathogens.^[^ ^183^ ^]^ Tumor progression locus 2 also vital in regulating inflammatory responses when Schistosoma mansoni infection occurs, facilitating lipolysis and M2 macrophage activation.^[^ ^184^ ^]^ In brief, the metabolic changes in macrophages regulated by microbes also play a significant role in tumor progression; the relationship of mutual symbiosis between microbes and macrophages preserves the dynamic stability of TME (Figure 7B–D).

## 5 CONCLUSIONS AND FUTURE PERSPECTIVES

This accumulation of data strengthens the evidence that TDEVs can deliver intracellular contents to healthy cells, notably the transportation of malignant factors that can strongly support tumor growth, metastasis, drug resistance, and other processes.^[^ ^185^ ^]^ However, few studies have focused on the regulation and feedback among tumor cells, TAMs, and the microbiota. In addition, the exact mechanisms remain unclear. In this review, a general field of vision on the cell‐to‐cell interactions was provided primarily consisting of three portions: (1) Tumor recruits accelerate the phenotypic transformation of macrophages through EVs, and the defected macrophages promote tumor progression through malignant feedback. (2) Tumor recruits that transfer malignant characteristics to microbes via EVs, and the tumor also facilitates the survival, migration, and replication of microbes. In turn, the microbes generate tumor‐promoting feedback to favor tumor progression. (3) Hijacking effects that lead to the reciprocal coexistence of macrophages and the microbiota. Therefore, the components in the TME that can interact with each other and form a dynamic loop that may contribute to tumor progression were highlighted. However, there are undoubtedly many more unknown interplays between tumor cells and other factors, and more effort is needed to reveal them.

Tumor therapy based on malignant loop intervention may be promising; TAMs are an important part of this loop. Studies have shown that TAMs are not only a unique M1 or M2‐like cell population, but they may also possess both M1‐ and M2‐like phenotypes.^[^ ^18^ ^]^ Therefore, the widely proposed use of TAMs remodeling and repolarization requires more research support. Currently, the studies based on phagocytic checkpoint and phagocytic activation are increasingly in the spotlight (Table 2). Macrophages may enhance their phagocytic capability through the phagocytic checkpoint and phagocytic activation like the axis regulation of CD47‐signal regulatory protein alpha PD‐1‐PD‐L1, or MHCI‐leukocyte immunoglobulin‐like receptor B1.^[^ ^186^ ^]^ More tumor cells and tumor‐associated microbiota can be eliminated subsequently.

**TABLE 2:** Clinical trials of tumor therapy based on TAM targeting

**Therapy pathway**	**TAM‐targeted factor**	**Drug**	**Combined therapy**	**Tumor category**	**Clinical trials**
TAM recruitment	CCL2	CNTO888	Carlumab	Prostate cancer	Phase2/NCT00992186
			DOXIL®/Caelyx® doxorubicin HCl liposome/gemcitabine/Paclitaxel/carboplatin/docetaxel	Cancer	Phase1/NCT01204996
	CCR2	PF‐04136309	Nab‐paclitaxel/Gemcitabine	Metastatic pancreatic ductal adenocarcinoma	Phase2/NCT02732938
			Placebo	Osteoarthritis, knee	Phase2/NCT00689273
			—	Healthy	Phase1/NCT02598206
			Oxaliplatin/Irinotecan/Leucovorin/Fluorouracil	Pancreatic neoplasms	Phase1/NCT01413022
	CCR2/CCR5	BMS‐813160	Nivolumab/Gemcitabine/Nab‐paclitaxel	Pancreatic ductal adenocarcinoma	Phase2/NCT03496662
			Stereotactic body radiation (SBRT) Nivolumab/GVAX	Advanced PDAC	Phase2/NCT03767582
			Nivolumab/MS‐986253	NSCLC/Hepatocellular carcinoma	Phase2/NCT04123379
			Nivolumab/Ipilimumab/Relatlimab BMS‐986205	Advanced cancer	Phase2/NCT02996110
TAM survival	CSF‐1R	RG7155	Atezolizumab	Solid tumors	Phase1/NCT02323191
		IMC‐CS4	Cyclophosphamide/GVAX/Pembrolizumab	Pancreatic cancer	Phase1/NCT03153410
		FPA008	BMS‐936558	Advanced solid tumors/Head and neck cancer/Pancreatic cancer/Ovarian cancer/Renal cell carcinoma/Malignant glioma	Phase1/NCT02526017
		Nivolumab	Relatlimab/Cabiralizumab/Ipilimumab/IDO1 inhibitor/Radiation therapy	Advanced cancer	Phase1/NCT03335540
		PLX3397	Paclitaxel	Solid tumors	Phase1/NCT01525602
		RG7155	Atezolizumab	Diffuse‐type giant cell tumor	Phase1/NCT02323191
TAM reprogramming/functional recovery	CD40	Selicrelumab	Atezolizumab	Solid tumors	Phase1/NCT02304393
		Atezolizumab	Capecitabine	Triple‐negative breast cancer	Phase2/NCT03424005
		Gemcitabine	Nab‐Paclitaxel	Pancreatic adenocarcinoma	Phase1/NCT03193190
		Regorafenib	Atezolizumab	Colorectal cancer	Phase2/NCT03555149
	CD47	Hu5F9‐G4	—	Solid tumors	Phase/NCT02216409
		Magrolimab	Azacitidine	Hematological malignancies	Phase1/NCT03248479
		TTI‐621	Rituximab/Nivolumab	Hematologic malignancies/Solid tumors	Phase1/NCT02663518
			PD‐1/PD‐L1 Inhibitor/pegylated interferon‐α2a/radiation/T‐Vec	Solid tumors/Melanoma/Merkel‐cell carcinoma/Squamous cell carcinoma/Breast carcinoma	Phase1/NCT02890368
			Doxorubicin	Leiomyosarcoma	Phase2/NCT04996004
			Pembrolizumab/Cisplatin/Carboplatin/5FU	Head and neck cancer	Phase2/NCT04675333
			Pembrolizumab	Head and neck cancer	Phase2/NCT04675294
		IBI188	Rituximab	Advanced malignancies	Phase1/NCT03717103

Not only miRNAs, but TDEVs also contain RNA, DNA, proteins, and metabolites from tumor cells, which carry excellent biological information of tumors. They are essential messengers in the malignant loop that may enter the local microenvironment or the systemic circulation after being secreted. This makes them increasingly recognized as promising circulating biomarkers.^[^ ^187^ ^]^ In addition, there is the potential to develop EV‐targeted antibodies in future studies.

Strands of evidence have uncovered cancer type‐specific signatures of microbiota,^[^ ^188^ ^]^ and substantial attention has already been paid to the microbiota and gastrointestinal tumors as a result of a much greater abundance of microbiota in the gut.^[^ ^189^ ^]^ However, microbial tumorigenesis is not entirely determined by abundance, and the tremendous biotechnological advances, such as, 16SrRNA, offer us new insights into the relationship between the microbiota and other tumors.^[^ ^190^ ^]^ Thus, it is believed that the malignant loop is universal in oncology. Many pathogens are highly related to tumors and even migrate with tumor cells to para‐cancerous tissue and distal areas.^[^ ^116^ ^]^ On the other hand, some probiotics contribute to the prevention and treatment of tumors. Some microbial metabolites, such as butyrate, short‐chain fatty acids, and inosine, can promote the efficacy of chemotherapeutic drugs by regulating the function of CD8^+^ T cells in the TME.^[^ ^191^, ^192^, ^193^ ^]^ Besides, researchers also proved that *Bifidobacterium* could enhance the function and metabolism of intestinal Treg cells, regulating the disordered microbial composition, alleviating the intestinal inflammation induced by checkpoint blockade immunotherapy.^[^ ^194^ ^]^ Consequently, Breaking the malignant loop among tumor cells, TAMs, and the microbiota may engender therapeutic benefits in clinical tumor intervention.

## CONFLICT OF INTEREST

There are no known conflicts of interest associated with this manuscript.
